# Prevalence of Dysmagnesemia among Patients with Diabetes Mellitus and the Associated Health Outcomes: A Cross-Sectional Study

**DOI:** 10.3390/biomedicines12051068

**Published:** 2024-05-12

**Authors:** Salwa Al Harasi, Juhaina Salim Al-Maqbali, Henrik Falhammar, Ali Al-Mamari, Abdullah Al Futisi, Ahmed Al-Farqani, Suneel Kumar, Alaa Osman, Sulaiman Al Riyami, Nafila Al Riyami, Qatiba Al Farai, Hiba Al Alawi, Abdullah M. Al Alawi

**Affiliations:** 1 Internal Medicine Residency Training Program, Oman Medical Specialty Board, Muscat 130, Oman; salwadoctor0@gmail.com; 2 Department of Pharmacology and Clinical Pharmacy, College of Medicine and Health Science, Sultan Qaboos University, Muscat 123, Oman; jsmm14@gmail.com; 3 Department of Pharmacy, Sultan Qaboos University Hospital, Muscat 123, Oman; 4 Department of Endocrinology, Karolinska University Hospital, 17165 Stockholm, Sweden; henrik.falhammar@ki.se; 5 Department of Molecular Medicine and Surgery, Karolinska Institute, 17177 Stockholm, Sweden; 6 Department of Medicine, Sultan Qaboos University Hospital, Muscat 123, Oman; amamari@squ.edu.om (A.A.-M.); futaisi@squ.edu.om (A.A.F.); farkani@squ.edu.om (A.A.-F.); drsk@squ.edu.om (S.K.); a.salah@squ.edu.om (A.O.); 7 Department of Biochemistry, Sultan Qaboos University Hospital, Muscat 123, Oman; suliemanm@squ.edu.om (S.A.R.); nafilariyami@gmail.com (N.A.R.); qatiba@squ.edu.om (Q.A.F.); hibah@squ.edu.om (H.A.A.)

**Keywords:** diabetes mellitus, magnesium deficiency, hypomagnesemia, hypermagnesemia, microvascular complications, chronic kidney disease

## Abstract

***Introduction:*** Magnesium is a vital intracellular cation crucial for over 320 enzymatic reactions related to energy metabolism, musculoskeletal function, and nucleic acid synthesis and plays a pivotal role in human physiology. This study aimed to explore the prevalence of dysmagnesemia in patients with diabetes mellitus and evaluate its correlations with glycemic control, medication use, and diabetic complications. ***Methods:*** A cross-sectional study was conducted at Sultan Qaboos University Hospital, including 316 patients aged 18 years or older with diabetes mellitus. Data included demographics, medical history, medications, and biochemical parameters. Serum total magnesium concentrations were measured, and dysmagnesemia was defined as magnesium ≤ 0.69 mmol/L for hypomagnesemia and ≥1.01 mmol/L for hypermagnesemia. ***Results:*** The prevalence of hypomagnesemia in patients with diabetes was 17.1% (95% CI: 13.3–21.7%), and hypermagnesemia was 4.1% (95% CI: 2.4–7.0%). Females were significantly overrepresented in the hypomagnesemia group, while the hypermagnesemia group showed a higher prevalence of hypertension, retinopathy, an increased albumin/creatinine ratio, chronic kidney disease (CKD), elevated creatinine levels, and a lower adjusted calcium concentration. The multinominal logistic regression exhibited that the female sex and higher serum-adjusted calcium were independent risk factors of hypomagnesemia. In contrast, the presence of hypertension, higher levels of albumin/creatinine ratio, and stage 5 CKD were independent risk factors of hypermagnesemia. ***Conclusions:*** Hypomagnesemia was common among patients with diabetes mellitus; however, hypermagnesemia was associated with microvascular complications.

## 1. Introduction

Magnesium ion is the second most important intracellular cation. There are approximately 21 to 28 g of magnesium in the human body, of which 99% is present in cells, and only 1% is present in the extracellular fluid [1,2]. Magnesium is essential as a cofactor for more than 320 enzymatic reactions involving energy metabolism, musculoskeletal function, and nucleic acid synthesis [3].

Humans obtain magnesium from a variety of sources: dairy products account for 20%, grain products for 18%, vegetables for 16%, meat, poultry, and fish for 15%, and legumes, nuts, and soy products for 13%. Additionally, drinking water can contribute up to 25% of an individual’s total magnesium intake [4].

In serum, magnesium presents in three forms: protein-bound (27%), ionized (65%), which is the biologically active form, and complexed (8%) with anions such as phosphate, bicarbonate, and citrate. Also, the serum magnesium concentration varies depending on the measurement method [5], but with atomic absorption methods, the normal magnesium range is about 0.7–1.0 mmol/L [1].

The homeostatic regulation of magnesium involves several hormones and vitamins, including parathormone (PTH), calcitonin, vitamin D, glucagon, antidiuretic hormone, and aldosterone. In addition, insulin is involved in transporting magnesium through the cellular membrane and intracellular supply [6]. Hypomagnesemia can cause several abnormalities to develop, including impaired glucose tolerance, insulin resistance (IR), and impaired lipid metabolism [7]. Moreover, hypomagnesemia is associated with poor health outcomes [8,9].

Hypomagnesemia in patients with diabetes mellitus (DM) is well established, which might be due to nutritional deficiencies, glucosuria-related hypermagnesiuria, and hyperinsulinemia-induced hypomagnesemia. The plasma magnesium concentration has been shown to be inversely related to insulin sensitivity [10]. The Atherosclerosis Risk in the Community (ARIC) study found that low serum magnesium concentration was a strong, independent predictor of incident type 2 diabetes (T2DM) [11]. Also, it was found that there was an inverse association between magnesium intake and the risk of developing T2DM among middle-aged women [12]. Another study found that ionized magnesium and intracellular free magnesium, but not total serum magnesium, were reduced in patients with T2DM compared to controls without T2DM [13].

Hypomagnesemia was found to be associated with an increased risk of diabetic retinopathy, diabetic nephropathy, and diabetic neuropathy [1,14]. It was estimated that the prevalence of hypomagnesemia in patients with T2DM ranged between 13.5 and 47.7% [15]. There was also a higher prevalence of hypomagnesemia (52%) in patients with diabetic nephropathy (DN) than in those without DN (22%). Previous studies have also shown that hypomagnesemia can affect the development and progression of DN [16].

Hypomagnesemia correlates with nerve conduction parameters in patients with T2DM, and abnormal magnesium status might explain the pathophysiologic features of diabetic peripheral neuropathy. In previous studies, serum magnesium correlated with signal amplitude, indicative of axonal function in peripheral neurons [7].

Another study investigated the association between serum trace elements, oxidative stress, HbA1c, growth stages, and insulin dose in younger patients with type 1 diabetes mellitus (T1DM), and it found that concentrations of serum magnesium were significantly lower in patients with T1DM compared to healthy individuals. However, there were no significant associations between serum magnesium concentrations, HbA1c levels, duration of T1DM, and insulin doses [17].

DM and its complications represent a substantial burden in the health care system worldwide. The projected rise in the global prevalence of DM has been estimated to be 55% between 2013 and 2035 [1]. Four out of five people with DM live in middle-income or low-income countries where resources are sparse [18]. The complications of DM are responsible for approximately 4 million deaths every year [7]. 

Studies examining hypomagnesemia in patients with diabetes mellitus (DM) have yielded conflicting results. Moreover, although recent research indicates an association between hypermagnesemia and poor health outcomes, this relationship has not been specifically studied in patients with DM before [9,19,20,21]. Thus, the objective of this study was to evaluate the prevalence of dysmagnesemia in patients with DM and establish correlations between dysmagnesemia, glycemic control, the number of DM medications, and the prevalence of diabetic complications.

## 2. Materials and Methods

### 2.1. Study Design, Setting, and Population

A cross-sectional study was conducted at Sultan Qaboos University Hospital (SQUH) from 1 August 2022 to 31 March 2023. SQUH, an academic tertiary hospital with a capacity of 500 beds, offers multispecialty care for inpatients and outpatients from all areas of Oman [22]. The endocrine outpatient clinic operates twice a week, with a total capacity of 60–80 patients per clinic.

Patients aged 18 years or older who visited the endocrine outpatient clinic for diabetes management were offered the opportunity to join the study. Interviews took place in the triage area, where informed consent was secured from either the patients themselves or their next of kin. Following consent, blood samples were collected in the phlebotomy room by a team of staff nurses and research assistants.

### 2.2. Data Collection

Relevant demographic data of patients, including age and sex, as well as medical history (such as hypertension, DM, chronic kidney disease (CKD), and heart failure (HF)), were collected. Medications known to potentially cause dysmagnesemia, such as loop diuretics, thiazides, and proton pump inhibitors (PPIs), were documented. Additionally, certain biochemical data were obtained, including vital electrolyte concentrations such as total calcium, potassium, and sodium, along with creatinine concentration and estimated glomerular filtration rate (eGFR), HbA1c, and albumin/creatinine ratio (ACR). Oral hypoglycemic medications and insulin doses were also collected. Information regarding retinopathy was ascertained by reviewing patients’ electronic medical records. The total concentration of magnesium was ascertained through a colorimetric endpoint reaction between magnesium and xylidyl blue in an alkaline solution. This analysis was conducted using the Roche Cobas modular analyzer in the Biochemistry Department at SQUH.

### 2.3. Definitions

According to the reference range established by the Biochemistry Department of our hospital, hypomagnesemia is defined as a magnesium concentration of ≤0.69 mmol/L, while hypermagnesemia is identified when the magnesium concentration is ≥1.01 mmol/L. Dysmagnesemia includes both conditions, hypomagnesemia and hypermagnesemia.

### 2.4. Sample Size

It was estimated that the prevalence of hypomagnesemia in patients with T2DM ranges between 13.5 and 47.7% [15]. We hypothesize that approximately 25% of patients with DM in our setting would have hypomagnesemia, a proportion we deem clinically significant in relation to diabetic complications. Consequently, the estimated sample size was set at 285 patients to achieve a 95% confidence concentration and 80% power to address the study’s primary objective. To accommodate potential missing information and loss to follow-up, the study’s sample size was increased by 10%, resulting in a total requirement of 313 patients.

### 2.5. Statistical Analysis

Categorical variables were presented in frequencies and percentages. Mean and standard deviation (SD) were used to describe normally distributed variables, while the median and interquartile ranges (IQRs) were utilized for continuously abnormally distributed variables. The chi-square test was employed to ascertain relationships between categorical variables and different magnesium concentration groups, with Fisher’s exact test applied when cells had fewer than five observations. Student’s *t*-test or the Kruskal–Wallis test was conducted to explore the relationship between normally or abnormally distributed variables and various magnesium concentration groups. All variables with a significance concentration (p) less than 0.05 were included in the backward stepwise multinominal logistic regression to identify potential independent factors associated with hypomagnesemia concentration compared to hypermagnesemia and normomagnesemia together and independent factors associated with hypermagnesemia compared to hypomagnesemia and normomagnesemia together. The two-tailed significance concentration was set at *p* < 0.05. Statistical analysis was performed using STATA version 17.0 (StataCorp, 1985–2021, Stata Statistical Software version 17.0, College Station, TX, USA).

## 3. Results

Throughout the study duration, a total of 2440 patients with DM were identified from the endocrinology clinic appointments list. Excluding those under 18 years old, n = 316 individuals were randomly recruited from the outpatient clinic and agreed to participate in the study.

In this cohort, 171 individuals (54.1%) were females, with a median age of 63 years (IQR 55–68) and a median DM duration of 15 years (IQR 10–22). The prevalence of hypomagnesemia among patients with DM was 17.1% (95% CI: 13.3–21.7%), while the prevalence of hypermagnesemia was 4.1% (95% CI: 2.4–7.0%).

As shown in Table 1, females with DM were significantly overrepresented in the hypomagnesemia group. In contrast, the hypermagnesemia group demonstrated a higher prevalence of the following: presence of hypertension, retinopathy, increased urine albumin/creatinine ratio, any stage of CKD, stages 4 and 5 CKD, elevated creatinine concentrations, reduced eGFR, and lower adjusted calcium concentration. While HbA1c did not differ with dysmagnesemia in our cohort, the hypermagnesemia group showed a higher prevalence of both long-term microvascular complications of DM, such as diabetic nephropathy and diabetic retinopathy. Moreover, the duration of DM was found to be longer in the hypermagnesemia group.

The effect of diabetes medications and other medications is shown in Table 2. Patients using only one oral hyperglycemic agent (OHA) had a better chance of having normomagnesemia or experiencing a mildly elevated magnesium concentration. As the number of OHAs increased to two agents, patients were more in the hypomagnesemia group. However, patients with three OHAs were more in the normomagnesemia group. Patients on frusemide were more common in the hypermagnesemia group.

As shown in Table 3, the backward stepwise multinominal logistic regression analysis for different groups of magnesium concentrations identified independent risk factors for the incidence of hypomagnesemia when compared with normomagnesemia using the adjusted relative risk ratio (aRRR), including the following: being a female (aRRR: 2.46, 95% CI: 1.283–4.722, *p* < 0.01) and higher serum-adjusted calcium (aRRR: 44.22, 95% CI: 2.644–739.457, *p* < 0.01). Additionally, the model identified independent risk factors for the incidence of hypermagnesemia when compared with normomagnesemia, including the following: being a case of hypertension (aRRR: 17.55, 95% CI: 1.204–255.652, *p* < 0.01), stage 5 CKD (aRRR: 25.21, 95% CI: 4.362–145.717, *p* < 0.01), having higher albumin/creatinine ratio (aRRR: 1.01, 95% CI: 1.003–1.009, *p* < 0.01), and on one OHA (aRRR: 8.53, 95% CI: 1.817–40.037, *p* < 0.01).

## 4. Discussion

This cross-sectional study aimed to explore the relationship between dysmagnesemia and diabetes control, as well as health outcomes. It stands out as one of the few studies to investigate the prevalence of both hypermagnesemia and hypomagnesemia in outpatient individuals with diabetes mellitus, featuring a relatively large sample size. The prevalence of hypomagnesemia was 17.1%, and the prevalence of hypermagnesemia was 4.1%. Unlike previous studies, hypermagnesemia was prevalent in our patients with DM. Moreover, this study investigated the association between dysmagnesemia and diabetic outcomes and was able to identify independent factors, including female sex for hypomagnesemia and other independent factors for hypermagnesemia, such as low adjusted calcium concentrations, retinopathy, and nephropathy.

Overall, the reported prevalence of hypomagnesemia in T2DM ranges between 13.5% and 47.7% [15,23]. Moreover, studies from Saudi Arabia (n = 285, n = 62) found that 28.4–29.0% of the screened patients with T2DM had hypomagnesemia [24,25]. Furthermore, an Indian study including 100 patients reported a 30% prevalence of hypomagnesemia in patients with T2DM [26]. This variation could be related to differences in patient characteristics, the use of different cutoff values to define hypomagnesemia, differences in diabetic control, the presence of CKD, dietary habits, and the duration of DM. It is worth mentioning that the prevalence of CKD in previous studies was around 30% compared to 50% in this current study. The high prevalence of CKD in our cohort and the long duration of DM (15 years) might contribute to a prevalence of hypomagnesemia in the lower range compared to other studies [24,25].

Previous studies have reported that patients with T2DM and hypomagnesemia have higher HbA1c concentrations [24]. Diabetes is frequently associated with both extracellular and intracellular magnesium depletion [27]. Epidemiologic studies have found a high prevalence of hypomagnesemia in subjects with T2DM, especially in those with poor glycemic control [28]. Among the mechanisms that may contribute to magnesium depletion in diabetes, the most significant factors are low magnesium intake and increased urinary magnesium loss. Diabetes is associated with renal calcium and magnesium wasting, although the molecular mechanisms underlying these defects remain unknown [29].

Although HbA1c was not associated with hypomagnesemia in our study, we found an association between the number of OHAs and hypomagnesemia. Moreover, we found that more than 50% of included patients had CKD, which might contribute to impairment of magnesium excretion. Thus, the majority of patients with hypermagnesemia in the present study had low eGFR.

It has been found that several medications are associated with hypomagnesemia, including proton pump inhibitors (PPIs) and diuretics (loop- and thiazide-like diuretics). PPIs are commonly used worldwide, with or without a prescription, for the treatment of acid-related disorders [30]. Hypokalemia and hypomagnesemia are two metabolic alterations that are associated with long-term thiazide therapy [31]. In our study, 19.6% of patients were on PPIs, 7% on furosemide, and 11.1% on thiazides, but we could not establish a relationship between the use of PPIs, furosemide, and thiazides and hypomagnesemia. This is likely due to the nature of the study design and the effect of the long duration of diabetes among the study’s patients. However, a meta-analysis of observational studies found an association between hypomagnesemia and the use of PPIs, with an overall 1.43-fold increased risk of hypomagnesemia compared to those who did not use PPIs [30].

Magnesium deficiency has been implicated as a novel factor in the pathogenesis of many diabetic complications. Hypomagnesemia can be both a consequence and a cause of diabetic complications [32]. Our population with DM and hypermagnesemia had a high prevalence of retinopathy and nephropathy, as mentioned previously. The latter could be explained by the impaired magnesium excretion, which tends to occur in patients with severe CKD.

We found that females were significantly overrepresented in the hypomagnesemia group among patients with diabetes. In the Women’s Health Study, a cohort of 39,345 U.S. women aged more than 45 years with no previous history of cardiovascular disease, cancer, or T2DM completed a semiquantitative food frequency questionnaire and were followed for an average of 6 years. There was an inverse association between magnesium intake and risk of T2DM, independent of age and BMI [12].

In a population-based study (n = 1558), the prevalence of hypermagnesemia was estimated to be around 3.0% [33]. Among hospitalized patients, the prevalence of hypermagnesemia varied between 5.7% and 9.3% [34]. Approximately 10% to 15% of hospitalized patients with CKD are estimated to develop hypermagnesemia due to reduced renal magnesium excretion [35]. Hypermagnesemia among hospitalized patients has been linked to adverse health outcomes, including increased in-hospital mortality and one-year mortality [21].

In an outpatient setting, hypermagnesemia has been insufficiently studied. Our study offers new insights, suggesting that hypermagnesemia is associated with diabetic nephropathy and retinopathy. Additionally, hypermagnesemia correlated with an increased duration of diabetes.

This study has several strengths, such as a relatively large sample size, data that were prospectively collected, and the evaluation of both hypomagnesemia and hypermagnesemia. Furthermore, it sought to establish connections between dysmagnesemia and various risk factors and outcomes in individuals with DM. Nonetheless, the study faces certain limitations. Participant recruitment occurred over a brief period and exclusively from a specialized endocrinology outpatient clinic within a tertiary healthcare facility, which predominantly oversees complex cases with extended durations of diabetes. These conditions may restrict the generalizability of the findings. Moreover, the study assessed total magnesium concentrations instead of focusing on ionized magnesium, the biologically active form [36].

## 5. Conclusions

Hypomagnesemia was common among patients with diabetes mellitus. However, hypermagnesemia was associated with microvascular complications. Future studies on the role of magnesium supplementation in the prevention of T2DM and its complications, as well as similar studies on ionized magnesium concentrations in patients with DM, are recommended.

## Figures and Tables

**Table 1:** Characteristics of patients with diabetes mellitus classified according to total magnesium concentration.

Characteristic n (%) Unless Specified Otherwise	Total 316 (100%)	≤0.69 mmol/L 54 (17.1%)	0.70–1.00 mmol/L 249 (78.8%)	≥1.01 mmol/L 13 (4.1%)	*p*-Value
Females	171 (54.1%)	39 (72.2%)	127 (51.0%)	5 (38.5%)	<0.01
Age (years)	63 (55–68)	60 (43–69)	56 (40–66)	63 (55–68)	0.30
BMI (kg/m^2^)	29 (25–36)	29 (25–33)	28 (25–34)	27 (25–36)	0.98
Alcohol consumption	2 (0.6%)	0 (0%)	1 (0.4%)	1 (7.8%)	0.11
Smoking	6 (1.9%)	0 (0%)	5 (2.0%)	1 (7.7%)	0.19
DM Medical History					
Type 1 DM	71 (22.5%)	9 (16.7%)	60 (24.1%)	2 (15.4%)	0.47
Type 2 DM	245 (77.5%)	45 (83.3%)	189 (75.9%)	11 (84.6%)	0.41
Duration of DM diagnosis (years)	15 (10–22)	12 (9–19)	15 (11–21)	24 (23–27)	<0.01
HbA1c (%)	8.0 (7.7–8.7)	8.1 (6.8–10.0)	8.0 (6.8–9.2)	8.3 (7.7–8.7)	0.59
Hypertension	166 (52.5%)	28 (51.9%)	126 (50.6%)	12 (92.3%)	0.01
DM Microvascular Complication					
Diabetic nephropathy (albumin/creatinine ratio) (IQR)	2.1 (0.5–16.6)	2.5 (0.5–19.1)	2.1 (0.5–9.3)	140.0 (34.8–273.8)	<0.01
Diabetic retinopathy	53 (16.8%)	7 (13.0%)	39 (15.7%)	7 (53.9%)	<0.01
Other Medical History					
Chronic kidney disease (CKD)	170 (53.8%)	24 (44.4%)	134 (53.8%)	12 (92.3%)	<0.01
Stage 1 CKD	17 (5.4%)	2 (3.7%)	15 (6.0%)	0	0.88
Stage 2 CKD	90 (28.5%)	11 (20.4%)	78 (31.3%)	1 (7.7%)	0.06
Stage 3 CKD	43 (13.6%)	10 (18.5%)	32 (12.9%)	1 (7.7%)	0.53
Stage 4 CKD	13 (4.1%)	1 (1.9%)	6 (2.4%)	6 (46.2%)	<0.01
Stage 5 CKD	7 (2.2%)	0 (0%)	3 (1.2%)	4 (30.8%)	<0.01
Thyroid diseases	39 (12.3%)	4 (7.4%)	33 (13.3%)	2 (15.4%)	0.48
Heart failure	5 (1.6%)	0 (0%)	5 (2.0%)	0 (0%)	0.67
Ischemic heart disease	26 (8.2%)	2 (3.7%)	23 (9.2%)	1 (7.7%)	0.36
Stroke	15 (4.8%)	3 (5.7%)	10 (4.0%)	2 (15.4%)	0.16
Dyslipidemia	108 (34.2%)	15 (27.8%)	87 (34.9%)	6 (46.2%)	0.39
Lung diseases	14 (4.4%)	4 (7.4%)	9 (3.6%)	1 (7.7%)	0.28
Liver diseases	15 (4.8%)	0 (0%)	14 (5.6%)	1 (7.7%)	0.11
Electrolytes					
Plasma albumin (mmol/L)	45 (42–46)	44 (42–46)	45 (42–46)	43 (40–44)	0.12
Serum-adjusted calcium (mmol/L)	2.4 (2.3–2.4)	2.4 (2.3–2.5)	2.4 (2.3–2.4)	2.3 (2.3–2.4)	<0.01
Serum phosphate (mmol/L)	1.18 (1.08–1.29)	1.14 (1.09–1.26)	1.18 (1.06–1.28)	1.32 (1.18–1.37)	0.10
Serum sodium (mmol/L)	138 (137–140)	138 (135–140)	138 (137–140)	138 (137–139)	0.36
Serum potassium (mmol/L)	4.6 (4.3–5)	4.5 (4.3–4.9)	4.6 (4.3–5)	4.8 (4.6–5.6)	0.28
Serum creatinine (mmol/L)	68 (57–89)	64 (52–86)	67 (58–86)	233 (172–462)	<0.01
Serum eGFR (ml/min/1.73 m^2^)	90 (70–90)	90 (67–90)	90 (74–90)	24 (11–25)	<0.01

**Table 2:** The effects of diabetes medications and other medications classified according to total magnesium concentration.

Characteristic n (%) Unless Specified Otherwise	Total 316 (100%)	≤0.69 mmol/L 54 (17.1%)	0.70–1.00 mmol/L 249 (78.8%)	≥1.01 mmol/L 13 (4.1%)	*p*-Value
Diabetes Medications					
Insulin	209 (66.1%)	36 (66.7%)	175 (63.1%)	12 (92.3%)	0.09
Insulin dose (unit)	66 (38–95)	51 (28–84)	70 (40–100)	60 (51–78)	0.22
GLP-1A	24 (7.6%)	3 (5.6%)	20 (8.0%)	1 (7.7%)	0.91
Oral hypoglycemic agents (OHAs)	111 (35.1%)	18 (33.3%)	92 (37.0%)	1 (7.7%)	0.09
1 OHA	88 (27.9%)	14 (25.9%)	65 (26.1%)	9 (69.2%)	<0.01
2 OHAs	92 (29.1%)	22 (40.7%)	69 (27.7%)	1 (7.7%)	0.04
3 OHAs	74 (23.4%)	6 (11.1%)	67 (26.9%)	1 (7.7%)	0.02
4 OHAs	53 (16.8%)	11 (20.4%)	41 (16.5%)	1 (7.7%)	0.53
OHAs + insulin	119 (37.7%)	22 (40.7%)	94 (37.8%)	3 (23.1%)	0.57
Other Medications					
Loop diuretics (frusemide)	22 (7.0%)	2 (3.7%)	14 (5.6%)	6 (46.2%)	<0.01
Thiazides (hydrochlorothiazide)	35 (11.1%)	8 (14.8%)	26 (10.4%)	1 (7.7%)	0.63
Thiazide-like diuretics (metolazone)	10 (3.2%)	4 (7.4%)	5 (2.0%)	1 (7.7%)	0.05
Proton pump inhibitors	62 (19.6%)	9 (16.7%)	50 (20.1%)	3 (23.1%)	0.81

**Table 3:** Backward stepwise multinominal logistic regression model for independent factors associated with the different groups of magnesium concentrations.

Outcome Tested in the Model	Independent Factors	aRRR * [95% CI]	*p*-Value	Overall *p*-Value
Hypomagnesemia ≤0.69 mmol/L vs. Normomagnesemia 0.70–1.00 mmol/L	Female	2.46 [1.283–4.722]	<0.01	<0.01
	Serum-adjusted calcium (mmol/L)	44.22 [2.644–739.457]	<0.01	
Hypermagnesemia ≥1.01 mmol/L vs. Normomagnesemia 0.70–1.00 mmol/L	Hypertension	17.55 [1.204 –255.652]	0.036	<0.01
	Albumin/creatinine ratio (IQR)	1.01 [1.003–1.009]	<0.01	
	Stage 5 CKD	25.21 [4.362–145.717]	<0.01	
	One OHA	8.53 [1.817–40.037]	<0.01	
Hypermagnesemia ≥1.01 mmol/L vs. Hypomagnesemia ≤0.69 mmol/L	Duration of DM diagnosis (years)	1.12 [ 1.035–1.211]	<0.01	<0.01
	Serum creatinine (mmol/L)	1.01 [ 1.002–1.020]	0.018	

* Backward stepwise multinominal logistic regression model for an adjusted relative risk ratio (aRRR) for the following factors significantly (*p* < 0.05) associated with hypomagnesemia in the univariate analysis that includes female, serum-adjusted calcium, serum estimated glomerular filtration rate (eGFR), presence of two oral hypoglycemic agents, duration of DM diagnosis, hypertension, albumin/creatinine ratio, chronic kidney disease (CKD), serum creatinine, presence of one oral hypoglycemic agents, and frusemide.

## Data Availability

The raw data supporting the conclusions of this article will be made available by the authors on request.
